# Inherent Surface Properties of Adsorbent-Free Ultrathin Bi_2_Se_3_ Topological Insulator Platelets

**DOI:** 10.1038/s41598-019-55646-1

**Published:** 2019-12-13

**Authors:** Blaž Belec, Katja Ferfolja, Tanja Goršak, Nina Kostevšek, Sandra Gardonio, Mattia Fanetti, Matjaz Valant

**Affiliations:** 1 Materials Research Laboratory, University of Nova Gorica, Vipavska 11c, 5270 Ajdovščina, Slovenia; 2 Department for Materials Synthesis, Jožef Stefan Institute, Jamova 39, 1000 Ljubljana, Slovenia; 3 Jožef Stefan International Postgraduate School, Jamova 39, 1000 Ljubljana, Slovenia; 4 Department for Nanostructured Materials, Jožef Stefan Institute, Jamova 39, 1000 Ljubljana, Slovenia; 5 Institute of Fundamental and Frontier Sciences, University of Electronic Science and Technology of China, 610054 Chengdu, China

**Keywords:** Materials for optics, Nanoscale materials, Chemistry, Inorganic chemistry

## Abstract

We report on a hydrothermal synthesis of hexagonal ultra-thin Bi_2_Se_3_ platelets, which was performed without any organic reactants. The synthesis resulted in the particles with a surface, clean of any organic adsorbents, which was confirmed with a high-resolution transmission electron microscopy, zeta-potential measurements and thermogravimetric measurements coupled with a mass spectroscopy. Due to the absence of the adsorbed organic layer on the Bi_2_Se_3_ platelet surface, we were able to measure their inherent surface and optical properties. So far this has not been possible as it has been believed that such hexagonal Bi_2_Se_3_ platelets can only be prepared by a solvothermal synthesis, for which it was unable to avoid the organic surface layer. Here we explain the mechanism behind the successful hydrothermal synthesis and show a striking difference in zeta potential behaviour and UV-vis absorption characteristics caused by the adsorbed layer. The surface of the hydrothermally synthesized Bi_2_Se_3_ platelets was so clean to enable the occurrence of the localized surface plasmon resonance due to the bulk and topological surface electronic states.

## Introduction

Topological insulators (TI) are a novel class of materials that recently have been intensively studied due to their attractive electron properties that result from metallic, linearly dispersing, spin-polarized surface states, widely known as topological surface states (TSS). The TI have a bulk band gap but, opposite to ordinary insulators, possess TSS on their surfaces that are robust to non-magnetic impurities and disorder because of the combined effect of time-reversal symmetry and spin-momentum locking^1–3^. These peculiar properties make the TI as very promising materials for novel applications like quantum computing^4–6^, THz detectors^7,8^, plasmonics^9–11^, spintronics^3,12,13^, and for medical diagnostics and treatment^14,15^.

Bi_2_Se_3_ is one of the most promising TI materials for the above mentioned applications, due to a narrow bulk band gap of 0.3 eV and the single Dirac cone at the Γ point of the Brillouin zone. Apart from its special electronic properties, Bi_2_Se_3_ displays a distinctive crystal structure. It crystallizes in a rhombohedral crystal structure, in which five covalently bonded atomic sheets (Se-Bi-Se-Bi-Se) form a quintuple layer arranged along the z-axis. The quintuple layers are linked together by weak van der Waals forces^16,17^. This distinctive feature makes the Bi_2_Se_3_ crystals to grow in a variety of different low-dimensional nanostructures^18–30^. From the viewpoint of applications (e.g. spintronic, quantum computing etc.), the most interesting are plate-like Bi_2_Se_3_ nanoparticles. The most suitable form for these applications would be oriented Bi_2_Se_3_ thin films prepared, for example, with a directed assembly of the plate-like Bi_2_Se_3_ crystallites by an electrophoretic deposition^31–34^. Due to its geometry, the Bi_2_Se_3_ platelets self-assemble with its large surfaces parallel to the substrate, forming thin film-like structures that can be used as building blocks in the heterostructures^35^.

Up till now, the most exploited method for the synthesis of the Bi_2_Se_3_ platelets is the solvothermal method^21,22,24–27,29,36^. However, a disadvantage of this method is in use of toxic, expensive organic solvents (e.g. ethylene-glycol, diethylene-glycol, dimethylfuran, ethyleamine, etc.) and capping agents (e.g. acetic acid, PVP, ascorbic acid, etc.), which raises environmental issues. But the most serious disadvantage of the solvothermal synthesis is in a fact that it yields particles coated with an adsorbed thin amorphous layer of the organic solvents or surfactants^37,38^. The formed organic layer is detrimental in the application of TI in various devices. It impedes a direct contact, required between TI and other materials in heterostructures that exploit the inherent surface electronic properties of TI^1–3,39–41^. Consequently, the solvothermally synthesized Bi_2_Se_3_ platelets cannot be used in such applications. It has already been pointed out that it would be essential to prepare the Bi_2_Se_3_ platelets without the presence of the organic solvents or surfactants^25^. One of the possible approaches is the hydrothermal synthesis. Despite a successful preparation of a variety of Bi_2_Se_3_ nanostructures with well-defined morphologies, there are no reports on a successful synthesis of the shape-specific product with only the hexagonal Bi_2_Se_3_ platelets, using the hydrothermal method, that would allow avoiding the presence of organic adsorbents. The samples prepared hydrothermally usually contain, besides the hexagonally shaped platelets, also rod-like or sheet-like particles^19,42^. Moreover, Ota *et al*.^42^ claimed that the monodispersed hexagonally shaped platelets could only be prepared by the solvothermal method.

As it is presented in this paper, we have successfully solved the problem that limits the application of the Bi_2_Se_3_ platelets in technology for years. Herein, we report a facile hydrothermal synthesis of the Bi_2_Se_3_ hexagonal platelets without using any organic reagents. In contrary to the previous report^42^, we demonstrated that the hexagonal platelets can be obtained hydrothermally by precise control of supersaturation. The obtained particles have a clean, non-modified surface, free of any adsorbed amorphous layer. Such particles expose the real inherent properties of the Bi_2_Se_3_ surface, which we have measured. As we show here, the difference in the surface and optical properties compared to the surfaces with the adsorbed amorphous layer is striking.

## Results and Discussion

**Figure 1:**
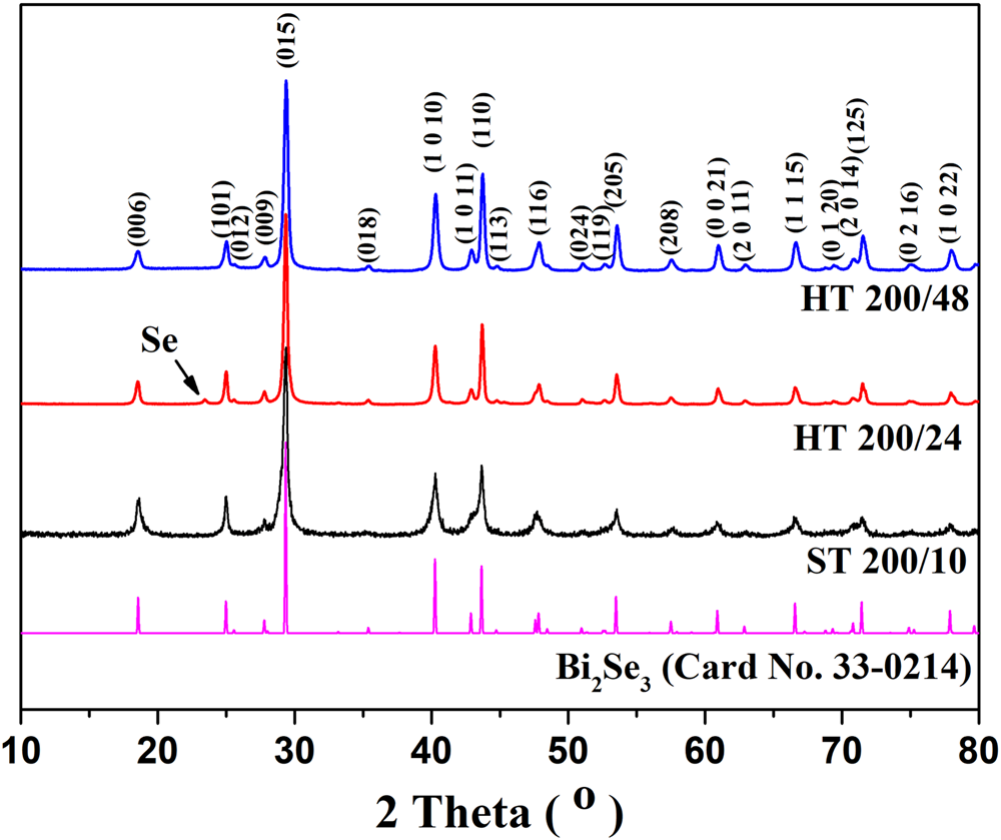
XRD patterns of Bi_2_Se_3_ platelets synthesized hydrothermally (HT) and solvothermally (ST) at 200 °C and different reaction times compared with the theoretical XRD pattern (Card No. 33-0214).

XRD patterns of the products, synthesized hydrothermally and solvothermally, are compared in Fig. 1. The diffraction patterns are very similar. The peaks can be indexed according to the Bi_2_Se_3_ rhombohedral structure (space group *R3m, JCPDS 33-0214*). The strong and sharp diffraction peaks indicate that Bi_2_Se_3_ is well crystallized. However, in the case of ***HT 200/24***, an additional peak of Se is observed. The Se peak is not observed anymore for the ***HT 200/48*** sample. The selenium could appear as a result of an incomplete reaction between bismuth and selenium, due to the short reaction time.

**Figure 2:**
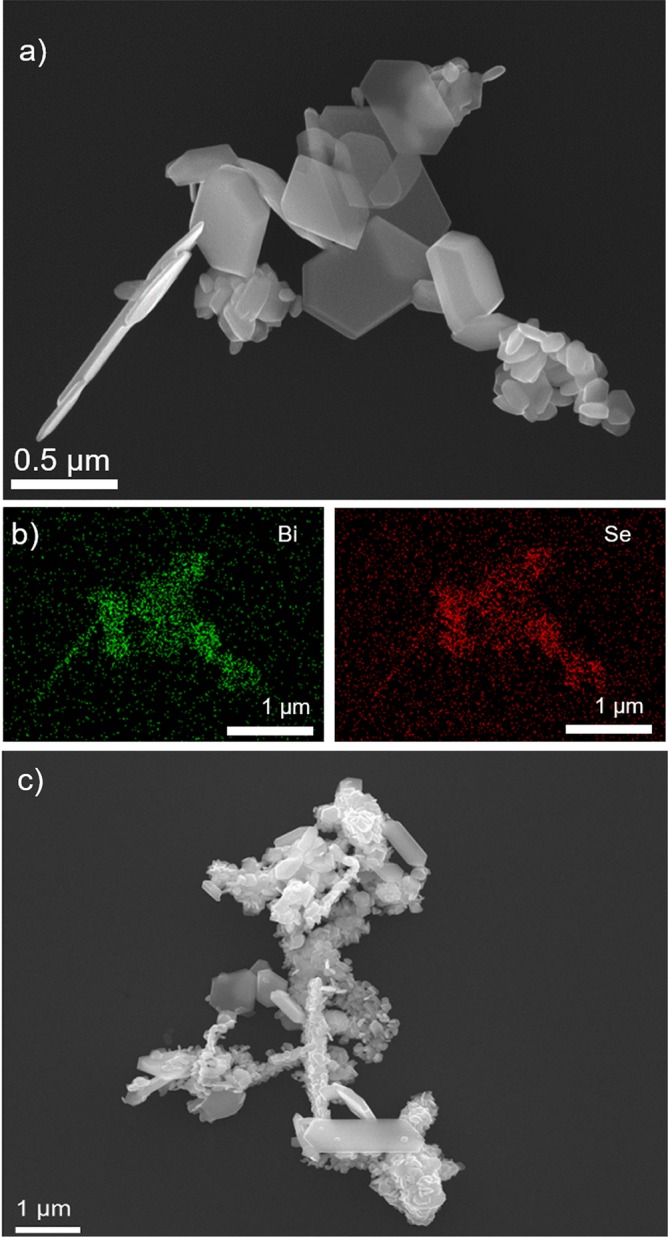
(**a**) SEM image of hydrothermally synthesized Bi_2_Se_3_ particles at 200 °C after 48 h with (**b**) corresponding EDXS elemental mapping and (**c**) SEM image of hydrothermally synthesized Bi_2_Se_3_ particles prepared at 200 °C after 24 h.

Figure 2a shows an SEM image of the hydrothermally synthesized product ***HT 200/48***. The product contains the hexagonally shaped platelets with a broad size distribution ranging from several tens of nanometres to a micrometre in diameter. The EDXS mapping (Fig. 2b) shows an increased signal of both elements, Bi and Se, over the region where the platelets are positioned, while the EDXS quantitative analysis on >100 platelets gave an atomic ratio Bi/Se = 0.66 ± 0.02, which is in a good agreement with the Bi_2_Se_3_ stoichiometry.

The literature reports claim that the reaction product with only hexagonally shaped Bi_2_Se_3_ platelets can only be obtained by the solvothermal method at temperatures above 150 °C^19,42^. It was explained that in the case of the hydrothermal synthesis, the reaction rate is much lower, which affects the product morphology. The slower reaction rate is a result of reduced ionic activity due to the more protic (polar) nature of water compared to the aprotic solvents (e.g. ethylene glycol). The reduced ionic activity promotes the growth of the particles in the preferential direction, forming particles with the rod-like morphology. According to the proposed growth mechanism^42^, the initial nanoparticles with a non-uniform sheet-like morphology transform into the rod-like particles at 150 °C^42^.

Here we show that contrary to the reported results, the hexagonally-shaped Bi_2_Se_3_ can be prepared with the hydrothermal synthesis at a somewhat higher temperature, 200 °C (Fig. 2a). Since the reaction rate is lower, due to the protic nature of water, the hydrothermal synthesis takes higher temperature and longer time compared to the solvothermal method. The SEM analysis revealed, that the Bi_2_Se_3_ platelets were formed already after 24 h, but some initial Se is still detected by XRD (Fig. 1); the reaction time is too short for the reaction between Bi and Se to be completed. Although the ***HT 200/24*** product is not single phase Bi_2_Se_3_, a majority of it are hexagonally shaped platelets (Fig. 2c).

Beside the ionic activity, which influences the reaction rate, the important parameter affecting the growth and morphology of particles is supersaturation (*σ*) of the reaction system^27,43^. According to the crystal growth theory, *σ* represents the driving force for crystal growth. Furthermore, the growth rate (*R*) of the crystal is linearly dependent on *σ*. Therefore, *R* linearly increases with *σ*^43^. In the case of the Bi_2_Se_3_ hydrothermal synthesis, *σ* can be controlled by the reduction rate of Se to Se^2-19,27,42^. The reduction rate can be controlled by a concentration of a reductive agent (hydrazine) and/or by NaOH concentration. NaOH, present in the reaction, provides OH^-^ ions that convert HSe^-^ into Se^2-^ in the final step of Se reduction process^42^. In the reported literature^19,42^, the authors had used a high concentration of reductive agent and/or NaOH, thus providing high *σ*. In our experiments, the concentration of the reductive agent was low, providing low *σ*. Moreover, to further decrease the hydrazine reducing capacity, we added 25 µL of 11 M HCl. According to Liu *et al*.^27^, the reductive capacity of the reductive agents can be decreased with a strong acid. Comparing the reaction conditions used in our experiments and those reported in the literature, we assume that the reason for obtaining the hexagonally shaped Bi_2_Se_3_ platelets with the hydrothermal method is in much lower supersaturation level.

**Figure 3:**
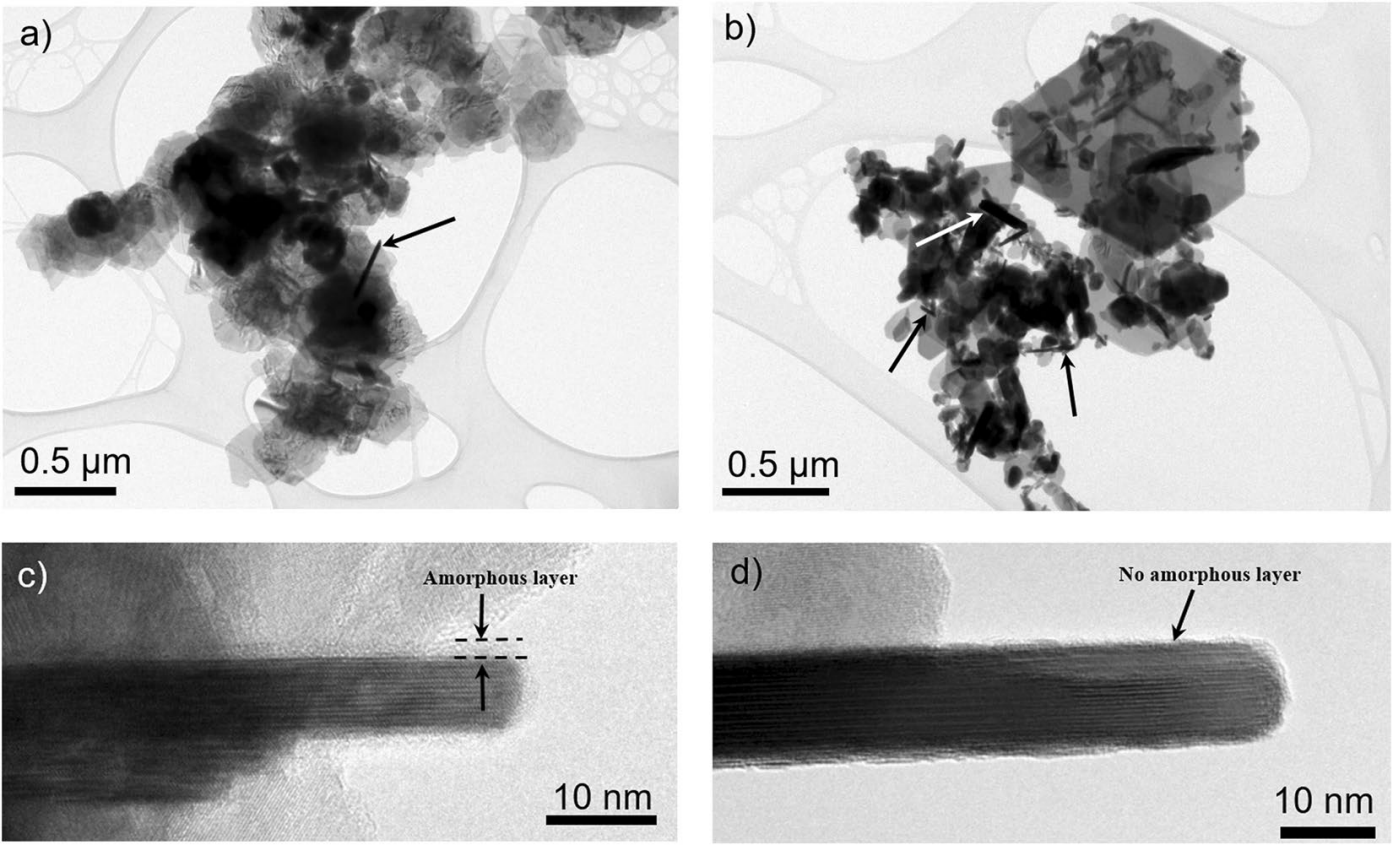
Representative TEM image of Bi_2_Se_3_ platelets synthesized (**a**) solvothermally at 200 °C for 10 h and (**b**) hydrothermally at 200 °C for 48 h. HR-TEM image of (**c**) solvothermally and (**d**) hydrothermally synthesized Bi_2_Se_3_ platelets orientated edge-on demonstrating the differences resulting from the different synthesis methods.

**Figure 4:**
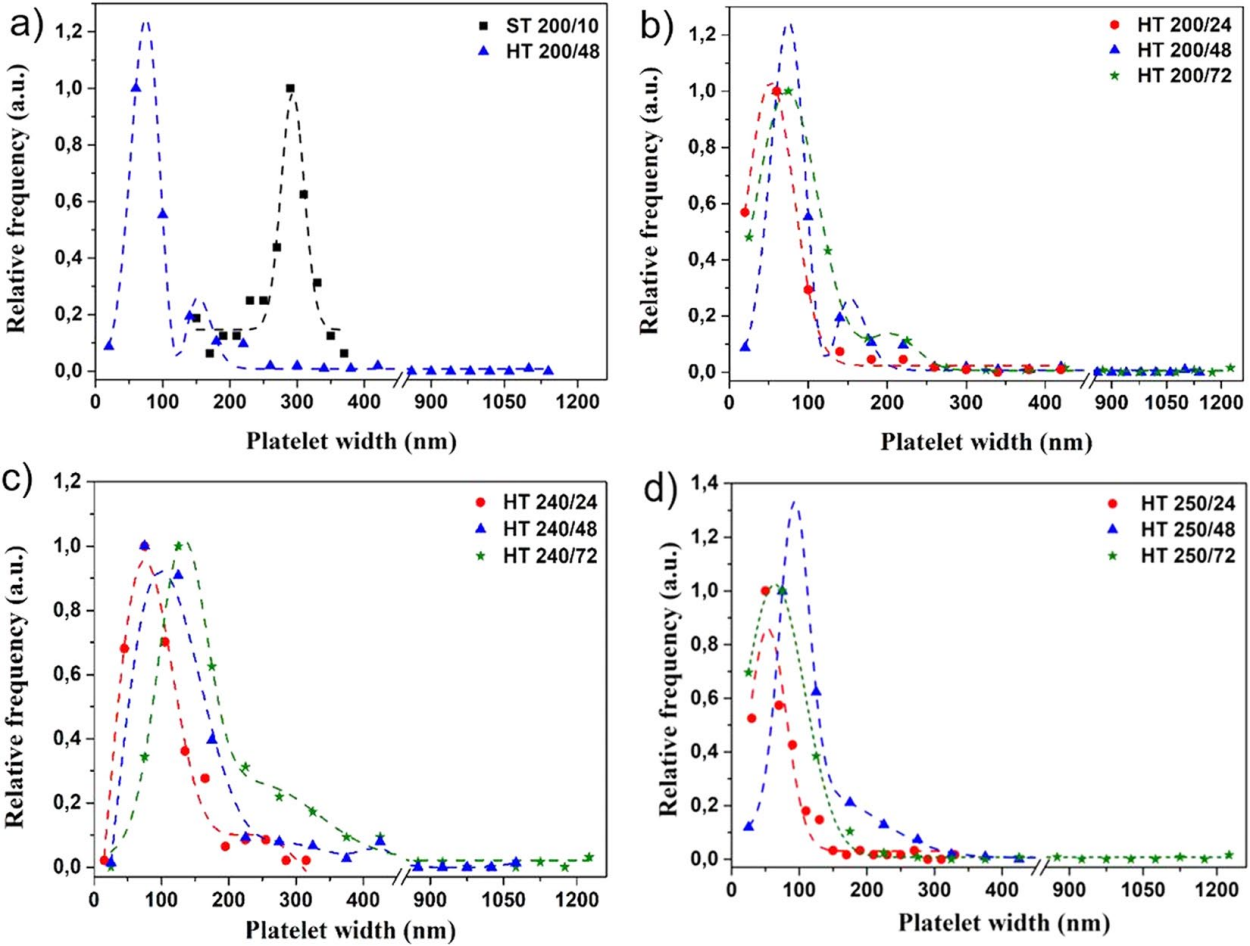
Bi_2_Se_3_ width measurements obtained from TEM images: a comparison between (**a**) the platelets synthesized by the solvothermal (ST) and hydrothermal (HT) method and a comparison between the hydrothermally synthesized platelets prepared at (**b**) 200 °C, (**c**) 240 °C and (**d**) 250 °C with different reaction time.

Figure 3a,b show representative TEM images of the Bi_2_Se_3_ platelets prepared solvothermally (***ST 200/10)*** and hydrothermally (***HT 200/48***), respectively. A comparison of platelet width distributions, measured from the TEM images and expressed as an equivalent diameter (Fig. 4a), revealed that the solvothermally synthesized platelets display a broad, monomodal size distribution with a diameter ranging from 200–400 nm. The hydrothermally synthesized platelets display a bimodal size distribution in a nanometre size regime, with diameters ranging from ≈ 20–100 nm and ≈150–250 nm. Moreover, the hydrothermal product also contains some larger platelets with a diameter up to ≈1.2 µm. The platelets’ thickness in both cases, the hydrothermal and solvothermal synthesis, was in a range from 10–20 nm, depending on the platelet diameter. However, due to the narrower size distribution in the case of the solvothermal synthesis, only the larger platelets, with a diameter close to 0.5 µm are 20 nm thick or more. A majority of the solvothermally prepared particles are 10 nm thick or even less. The difference in the size distribution can be ascribed to the use of different solvents. The hydrothermal performed synthesis results in the bimodal size distribution in the nanometre size regime and appearance of larger, micrometre sized platelets because the initially formed platelets grow with the secondary recrystallization (Ostwald ripening)^44–46^. In the case of the solvothermal method, the surfactants and shaping agents suppress the secondary recrystallization, therefore, block the excessive platelet growth. This leads to the narrower and monomodal size distribution^46^. We have also performed a synthesis with longer reaction time (up to 72 h) and/or at a higher temperature (240–250 °C). The size measurements of the hydrothermally synthesized Bi_2_Se_3_ platelets (>200 platelets per sample) indicate that there is not much difference between the sample prepared at prolonged time of 72 h at 200 ° compared to sample prepared at 48 h (Fig. 4b). The same is true also for the products obtained at 240 °C (Fig. 4c). All the products display a shoulder-like feature above 200 nm, indicating the bimodal size distribution of the platelets in the nanometre regime. For the samples prepared at 250 °C, only the sample treated for 48 h showed the broad shoulder-like feature, while samples treated for 24 or 72 h display the monomodal size distribution (Fig. 4d). However, with the increase in the reaction time and/or temperature, the average platelet size does not increase. This suggests that the system reached its thermodynamic equilibrium^44^.

The most important difference between the hydrothermally and solvothermally synthesized Bi_2_Se_3_ particles is shown on the HR-TEM images in Fig. 3c,d. The images display the platelets’ largest lattice periodicity of 0.95 nm, which corresponds to the (300) lattice planes of the rhombohedral Bi_2_Se_3_ structure^42,47^. The observation of the platelets orientated edge-on revealed that the platelets synthesized by the solvothermal method, are covered with an approximately 2 nm thick amorphous layer (marked on Fig. 3c). Contrary, the surface of the hydrothermally synthesized Bi_2_Se_3_ platelets is clean (Fig. 3d), without the presence of an amorphous layer. It is well known, that in the case of the solvothermal synthesis, the molecules of organic solvent or surfactant adsorb on the particles’ surface in the form of an amorphous layer^31,48–51^. In our case, the amorphous layer could consist of the adsorbed molecules of ethylene glycol or PVP. To provide additional proof for the presence of the organic layer adsorbed on the surface of the solvothermal Bi_2_Se_3_ platelets and its absence on the hydrothermally synthesized surfaces, zeta potential (ζ) measurements and thermogravimetry coupled with a mass spectroscopy (TG-MS) were performed.

**Figure 5:**
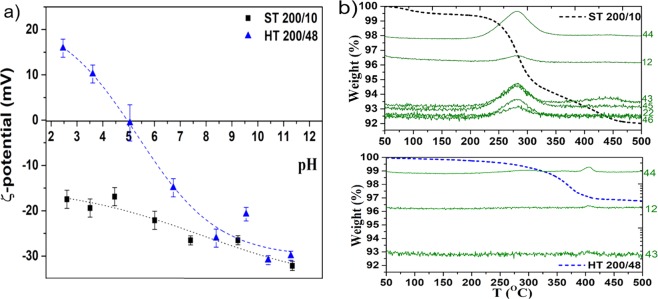
(**a**) Zeta-potential behavior of the hydrothermally (HT) and solvothermally (ST) synthesized Bi_2_Se_3_ platelets. (**b**) Mass loss during TG-MS experiment in nitrogen atmosphere for HT and ST samples heated from 50 to 500 °C. The green curves are intensities of chosen representative m/z fragments of evolved gases.

Figure 5a shows the zeta-potentials (ζ) of the platelets as a function of suspension pH. The Bi_2_Se_3_ layered structure terminates with Se^2-^ ions^25^. The main difference between both surfaces is that in the case of the solvothermally synthesized platelets, the surface charge results from the adsorbed organic molecules and not from the platelet surface directly. Considering that for unmodified particles the atoms on the particle surface dictate the surface charge^37^, we can explain the positive ζ of the hydrothermally synthesized platelets in acidic pH by adsorption of hydronium ion (H_3_O^+^). At the neutral and alkaline pH, the ζ values are negative due to the adsorption of hydroxyl ions (OH^-^) from water. For the solvothermally synthesized platelets, ζ is negative along the whole pH region between pH 2.5 and 11. This is due to the hydroxyl (-OH) groups of the ethylene glycol molecules (used as a solvent for the solvothermal synthesis) adsorbed onto the surface of the platelets during the reaction.

The presence of ethylene glycol on the surface of the solvothermally synthesized platelets was also confirmed by TG-MS. The TG curve of the solvothermally prepared Bi_2_Se_3_ platelets, shown in Fig. 5b, is very similar to those reported in literature^18,52^. During the sample heating to 500 °C in a N_2_ atmosphere, all together around 8% of the mass was lost. A significant mass loss can be observed at ≈ 250 °C. The MS curves corresponding to this weight loss (m/z = 42, 43, 45 and 46) belong to ethylene glycol fragments^53^. At the same temperature also the C (m/z = 12) and CO_2_ (m/z = 44) fragments are observed, which were also ascribed to the ethylene glycol. The third decrease in the mass can be seen at ≈ 440 °C and is accompanied by an increase of the most intensive fragment 43. This indicates on two fractions of ethylene glycol molecules, one directly bonded on the platelet surface with strong van der Waals forces and one bonded on longer distance with weaker electrostatic forces. On the contrary, the mass loss of the hydrothermally synthesized sample is smaller, only 3%. (Fig. 5b) The significant decrease in mass was observed only above 300 °C and was accompanied by an increase in the CO_2_ (m/z = 44) and C (m/z = 12) fragments. Since the hydrothermal synthesis was performed in the absence of any organic molecules, the observed CO_2_ and C fragments are due to CO_2_ surface adsorption during post-processing (e.g. powder washing, drying in air etc). No intensity increase of the glycol-specific fragments (e.g. m/z = 43) was observed. The results obtained with the TG-MS analysis additionally support the ζ-potential and TEM results and confirm that in the case of the solvothermal synthesis, the Bi_2_Se_3_ platelets are coated with the thin amorphous layer, which is ethylene glycol, while hydrothermal synthesized surfaces are free of the organics.

**Figure 6:**
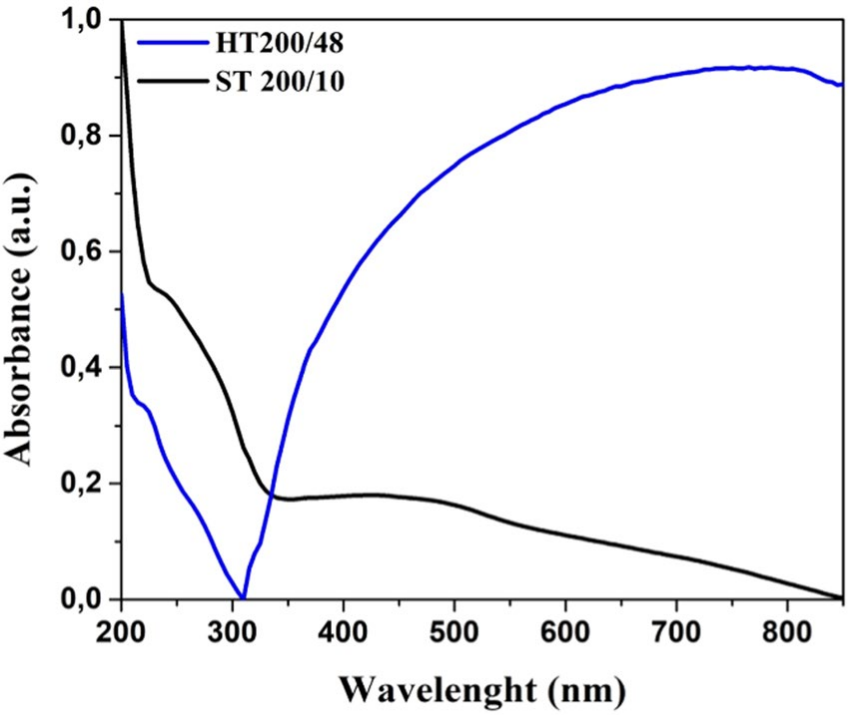
UV-vis absorption spectra of Bi_2_Se_3_ platelets synthesized hydrothermally (HT) and solvothermally (ST).

Figure 6 shows the UV-vis spectra of the Bi_2_Se_3_ platelets, synthesized solvothermally and hydrothermally, dispersed in water at room temperature. The solvothermaly prepared platelets display absorption peaks in the range of ≈210–300 and ≈350–550 nm, but towards the near infrared region the absorbance decreases without any absorption peaks. The behaviour is similar to what is reported in the literature^14,15,36,52,54^. Contrary, the absorption spectrum of the hydrothermally synthesized Bi_2_Se_3_ platelets is strikingly different. In the spectral region of ≈210–300 nm is very similar to what is observed for the solvothermally prepared platelets. However, at the higher wavelengths it displays an intense and broad adsorption peak that dominates the absorption over the entire measured range. A calculated plasmonic figure of merit predicts that the bulk intraband transition in Bi_2_Se_3_ lead to the plasmonic absorption in the range of 200–400 nm, while interband and intraband transitions involving TSS yield the plasmonic response in the range of 400–700 nm and above 1 μm, respectively^55^. By comparing our measurements and the calculation from ref. ^55^, the observed absorption features can be interpreted as due to a localized surface plasmon resonance (LSPR). Interestingly, only in the case of the platelets prepared with the hydrothermal method, the contribution due to TSS seems to be manifested.

Based on the microscopic characterization reported above, we propose that the quenching of the TSS plasmonic contribution in the case of the solvothermally prepared Bi_2_Se_3_ platelets, in the optical range, is due to the presence of the adsorbed organic layer. There are different reasons. One possibility is that the plasmon resonances due to TSS are shifted outside the visible range because of the organic layer like it is observed in metal nanoparticles^56^. The other reason can be that the adsorbed organic layer changes the structure of the top atomic layers of Bi_2_Se_3_ platelets. Therefore, the thickness of the intact quintuple layers of Bi_2_Se_3_ reduces, reaching the threshold thickness where there is the opening of the gap with the consequent disappearance of the TSS contribution to LSPR^57^.

## Conclusion

Many methods have been proposed to synthesize Bi_2_Se_3_ platelets. Among them, the solvothermal synthesis is the most exploited one^21,22,24–27,29^. Unfortunately, the disadvantage of this method is the use of organic reactants (solvents or capping agents), which tend to adsorb on the surface of the particles, as it was demonstrated in this work. The adsorbed amorphous layer limits the applicability of such Bi_2_Se_3_ platelets, since it appears to extinguish the plasmonic resonance involving TSS. The phenomena might be considered as an indirect signature of the TSS disruption of the TI, the property on which many applications rely^1–3,39–41^. As we demonstrated in this article, the hydrothermal synthesis without using any organic reactants can be applied for the synthesis of Bi_2_Se_3_ platelets despite the opposite claims reported in the literature. This can be achieved by careful control of supersaturation of reactant species. The synthesized product contained only hexagonally-shaped particles with a clean surface, free of any organic adsorbents. Due to this, we were the first to measure the true inherent surface and optical properties of the Bi_2_Se_3_ platelets. We demonstrated, that the properties are significantly affected by the adsorbed layer. We showed this on the case of the particle zeta potential and the UV-vis absorption characteristics. In contrary to the solvothermally synthesized particles, the TSS derived plasmonic features of the hydrothermally synthesized, are not disturbed due to the absence of the amorphous layer. Consequently, the inherent surface properties are manifested. Based on the comparison between experimental and calculated optical properties, we demonstrated that such surfaces display LSPR due to the electronic bulk states and TSS. The LSPR plays an important role in the photothermic effect of the Bi_2_Se_3_ platelets. Therefore, such Bi_2_Se_3_ platelets have great potential to be used as a photothermic conversion agent in cancer therapy^7,10,11,14^. Since the surface of the hydrothermally synthesized platelets is clean, they have great potential to be used as building blocks for devices where the intimate contact between the materials in the heterostructure must be assured.

## Methods

### Chemicals

Bismuth (III) nitrate pentahydrate (Bi(NO_3_)_3_·5H_2_O), bismuth (III) oxide (Bi_2_O_3_, 99.98%), selenium powder (Se, ≥99.5%), hydrochloric acid (HCl), hydrazine hydrate (N_2_H_4_·H_2_O, 35%), sodium hydroxide (NaOH), ethylene glycol (C_2_H_6_O_2_, 99%), polyvinilpyrolidone (PVP, MW = 8000) were used. All the chemicals were purchased from Alfa Aesar and were used without further purification.

For the synthesis of Bi_2_Se_3_ platelets by the hydrothermal method, the stoichiometric amounts of bismuth nitrate (1 mmol) and selenium (1.5 mmol) were dissolved in 20 mL of deionized water under vigorous stirring, followed by an addition of 11 M HCl (25 µL) and hydrazine (1.6 mL). The obtained grey slurry was sealed in a Teflon-lined autoclave and heated at various temperatures ranging from 200–250 °C for 24–72 hours. Afterwards, the autoclave was allowed to cool naturally to room temperature. Then the product was washed several times with deionized water. The platelets, synthesized with the hydrothermal method, are denoted as *“****HT T/t****”*, where *“****T****”* denotes the synthesis temperature in °C and *“****t****”* time of the reaction in hours.

To compare and evaluate the hydrothermal synthesis, the Bi_2_Se_3_ platelets were also prepared by the solvothermal method, according to the procedure described in ref. ^58^. In short, PVP and the stoichiometric amounts of Bi and Se were dissolved in 20 mL of ethylene glycol. The grey slurry was sealed in a Teflon-lined autoclave and heated to 200 °C for 10 h. When the reaction was completed, the product was washed several times with deionized water. The sample obtained with the solvothermal method is denoted as *“****ST 200/10****”*.

The synthesized product was characterized by an X-ray powder diffractometer Rigaku MiniFlex with Cu K*α* radiation (*λ*-1541 Å, 30 kV, 10 mA). The morphology and chemical composition of the platelets were analyzed by a scanning (SEM) and a transmission (TEM) electron microscopy. For the SEM analysis, the suspension containing the platelets was deposited on a Si-wafer and dried. The SEM analysis was performed using a field-emission scanning electron microscope (FESEM, JEOL JSM 7100 TTLS) equipped with an energy dispersive X-ray spectrometer (EDXS, Oxford X-Max80). For the TEM analysis, the platelets were suspended in ethanol and deposited on a copper-grid-supported lacey carbon film. The TEM analysis was performed using a field-emission electron microscope (JEOL JEM 2100UHR) operating at 200 kV and equipped with an Oxford X-Max80T energy dispersive X-ray spectroscopy detector (EDXS). The width of the platelets expressed as an equivalent diameter was determined from the TEM images, on which 200–300 platelets per sample were accounted for the statistic using Gatan Digital Micrograph Software. The obtained data, representing the frequency count of the size distribution, were fitted using the single or multiple peak Gaussian fit mode. Electro-chemical properties (ζ-potential) of the platelets dispersed in water were measured as a function of the suspension pH using a ZetaPALS instrument (Brookhaven Instruments Corporation). The pH of the aqueous suspension was adjusted with diluted hydrochloric acid and sodium hydroxide. The thermogravimetric analysis was performed by a TGA/DSC 2 (Mettler Toledo) thermal analyzer coupled with a mass spectrometer Thermostar 300 (Vacuum Pfeiffer). The samples were heated from 50 °C to 500 °C at 10 °C/min under a nitrogen atmosphere with a gas flow of 50 mL/min. The light absorption properties were analyzed with a classical UV-vis spectroscopy. The spectroscopy was performed by a PerkinElmer Lambda 950 spectrometer using a quartz cuvette with a size of 1 × 1 × 3 cm. A measurement range, λ, from 300 to 900 nm was used with a scanning rate of 1 nm/s. Prior to the measurements, the water suspension of the platelets was stabilized according to the ζ-potential and sonicated to break any possible agglomerates.
